# Therapeutic Uses of Glycerophosphates

**Published:** 1907-07

**Authors:** 


### Therapeutic Uses of Glycerophosphates.

C. D. F. Phillips, M.D., London, late Lecturer on Materia Medica at Westminister Hospital and Examiner in Aberdeen University, writing on "The Physiological Actions and Therapeutic Uses of Phosphorus and Some of its Compounds," says that the glycerophosphates are now extensively employed and appear to have a distinct sphere of usefulness. Discovered in the yolk of egg in 1846, they were but little known till 1893, when Robin drew attention to the large amount of partly oxidised phosphorus in the form of glycerophosphoric acid eliminated in the urine of some neurasthenics. He administered compounds of this acid and found that they increase the general metabolism of both organic and inorganic matter, principally the latter, as shown by the augmented excretion of urea, chlorides and sulphates, though not of uric acid or phosphates. They favor the current assimilation of albuminoid matters and moderate denutrition of the nervous system, adding its reconstruction by remaining in the body. They have a wide range of employment,—

chronic gout, diabetes, phthisis, Bright's and even Addison's disease with nutritive decay, to improve the vital powers rather than to directly combat such maladies; similarly, any nervous breakdown, as in the aged or after acute illness; chlorosis, rachitis; chronic dyspepsia, especially with lessened acidity (after appropriate treatment); and neuralgia, ataxia, sciatica, spermatorrhea and neurasthenia generally when marked by depression, headache and impaired mental and muscular strength. Robin said insomnia, palpitation and phenomena of excitement may be exaggerated (this is not Phillips's experience) and that the medication is not suitable in conditions of azoturia or when organic oxidations are above normal, or in mental disease or general paralysis. They are beneficial in muscular atrophy from various causes and in diphtheritic paralysis.

Harris pointed out that a priori it is probable that the glycerophosphates act as foods to the nervous system. They favor assimilation and metabolism. The best results are obtained in nervous exhaustion from overwork, in which they really have good effect.

Professor Wild, of Manchester, uses glycerophosphate of soda for weakness following influenza and for nervous exhaustion from overwork and Dreschfield finds it useful in many functional diseases.

